# Prioritizing involuntary immobility in climate policy and disaster planning

**DOI:** 10.1038/s41467-025-57679-9

**Published:** 2025-03-16

**Authors:** Lisa Thalheimer, Fabien Cottier, Andrew Kruczkiewicz, Carolynne Hultquist, Cascade Tuholske, Hélène Benveniste, Jan Freihardt, Mona Hemmati, Pui Man Kam, Narcisa G. Pricope, Jamon Van Den Hoek, Andrew Zimmer, Alex de Sherbinin, Radley M. Horton

**Affiliations:** 1 International Institute of Applied Systems Analysis, Laxenburg, Austria; 2 Institute for Environment and Human Security, United Nations University, Bonn, Germany; 3 Center for Integrated Earth System Information, Columbia Climate School, Columbia University, New York City, NY USA; 4 National Center for Disaster Preparedness, Columbia Climate School, Columbia University, New York, NY USA; 5 Faculty of Geo-information Science and Earth Observation, University of Twente, Enschede, The Netherlands; 6 School of Earth and Environment, University of Canterbury, Canterbury, New Zealand; 7 Department of Earth Sciences, Montana State University, Bozeman, MT USA; 8 Geospatial Core Facility, Montana State University, Bozeman, MT USA; 9 Department of Environmental Social Sciences, Doerr School of Sustainability, Stanford University, Stanford, CA USA; 10 Department of Mechanical and Process Engineering, ETH Zürich, Zürich, Switzerland; 11 Lamont-Doherty Earth Observatory, Columbia Climate School, Columbia University, New York City, NY USA; 12 Department of Environmental Systems Science, ETH Zürich, Zürich, Switzerland; 13 Department of Geosciences, Mississippi State University, Mississippi, MS USA; 14 College of Earth, Ocean, and Atmospheric Sciences, Oregon State University, Corvallis, OR USA; 15 Columbia Climate School, Columbia University, New York City, NY USA

**Keywords:** Natural hazards, Climate-change impacts, Climate change

## Abstract

Globally, populations are increasingly located in areas at high risk of climate change impacts. Some populations lack the agency to move out of harm’s way, leading to involuntary immobility. The climate risks these populations face are insufficiently addressed in climate policy and disaster planning. While policy and planning should be data-informed, the lack of appropriate data should not limit governments and institutions from taking action to reduce the risk of involuntary immobility. Incorporating involuntary immobility within the broader sustainable development goals of climate action and safe, orderly, and regular migration may substantially reduce the risk of involuntary immobility.

## Abstract (web summary)

Climate change and extreme weather events will influence migration patterns. However, it is important that our policies do not forget about those who are immobile to migration. This perspective explores different forms of involuntary immobility and how climate policy can address this.

## Introduction

Climate change impacts are unlikely to lead to mass migration and, in some contexts, can force people to stay in place. This situation is known as involuntary immobility, where people cannot move due to constraints such as climate stressors, poverty, legal barriers, conflict, or the intersection thereof^1–4^. Involuntary immobile populations are large and varied in their exposures and socioeconomic contexts and are primarily located in informal settlements, refugee camps, prisons, and conflict zones^5,6^. Most climate mobility research has focused on mobile populations, *de facto* excluding those unable or unwilling to move^5,7,8^. This omission is tied to the lack of political representation and difficulty observing immobility, which lacks distinct data of such “non-observations.” These diverse populations require tailored policies to reduce climate risk, ensure that their migration aspirations are met, and help reduce existing barriers to migration^9,10^. Recent scholarship has begun addressing involuntary immobility in the context of climate change but lacks systematized knowledge about its drivers^11–13^.

Mobility outcomes can be understood using the aspirations-capabilities framework, where aspirations refer to a person’s desire to move, and capabilities capture whether they can do so^7^. Those who wish to and can move experience voluntary mobility, while those who want to but cannot move are caught in involuntary immobility, sometimes referred to as "trapped populations"^11,14^. Among those who do not want to move, there’s a conceptual distinction between those who could move (voluntary immobility) and those who cannot (acquiescent immobility), although empirically distinguishing these two categories is challenging^11^.

Involuntary immobility is the result of a variety of intersecting factors^15^. These include, but are not limited to, economic, political, legal, social, and cultural obstacles, which collectively inhibit a person’s ability to move and to find shelter in perceived safer areas. Involuntary immobility is not an inherent characteristic of a population but is context-specific; for example, immobility might follow mobility when moving from a precarious place to the next, where immobility then occurs^16,17^. Involuntary immobility in the context of climate change is more than a data concern; it is a lens for guiding climate action and social justice to support vulnerable populations. It is crucial to consider people’s differing aspirations and capabilities regarding mobility in policies aimed at helping them adapt in situ or move to safer areas^13,18^.

Climate change and variability can influence various dimensions of involuntary immobility in increasingly interconnected ways^18,19^. For this Perspective, involuntary immobility in the context of climate change pertains to political, socioeconomic, and environmental contexts in which individuals, households, or communities face risks to their livelihoods due to exposure to slow-onset (e.g., droughts, sea level rise, soil salinization) or rapid-onset (e.g., flooding, landslides, heat waves) natural hazards. They are either unable to leave their residence despite a desire to do so or perceive that any such attempt would be unsuccessful or ineffective^10,20^. We argue that policies and services that can anticipate such changes can better support potentially immobile populations and mitigate the conditions driving such a shift in the context of climate change.

Building on a World Cafe, a large group exploratory dialog (see Methods), we develop a set of general principles by identifying the key socioeconomic drivers of involuntary immobility that make marginalized populations even more vulnerable to natural hazards and climate change impacts. To do so, we showcase the diverse drivers of involuntary immobility in the context of climate change through case studies. We conclude with research and policy implications for improving data collection strategies, ensuring knowledge co-production, and implementing pragmatic solutions that alleviate vulnerabilities to natural hazards faced by involuntarily immobile populations and integrate their aspirations.

## Drivers of involuntary immobility in the context of climate change

For both short- and long-term immobility, we apply a widely used multicausality conceptual framework approach^21^ focusing on economic and physical; political, legal and bureaucratic; and social and cultural drivers. In doing so, we seek to demonstrate how these three classes of drivers compound and contribute to the risk of involuntary immobility in the context of climate change, relying on illustrations from individual case studies.

### Economic and physical drivers

The classic perspective on migration has long documented that economic considerations are important constraints^22,23^. The upfront costs a migrant must pay to cover travel expenses exert a significant constraint on the ability to move, particularly over long distances and through regions with complex geographic and socioeconomic deterrents^24,25^. Relatively lower-income households face substantial financial challenges to pursue migration, even when across shorter distances^26,27^. Also, perceptions of current and future economic conditions can influence long-term mobility options^2^.

Financial constraints to migration can also be understood in the context of livelihood choices for individuals and households. Livelihood choices of smallholder farmers in Nepal demonstrate that international economic migration can be a higher-cost, higher-reward strategy compared to other local options, but projected climate-related reductions in agricultural income by mid-century are expected to constrain the ability of households with lower access to capital to choose migration as a livelihood strategy^2^. As another illustration, under future sea level change (SLC) scenarios, occurrences and experiences of flooding and associated damages from SLC are paradoxically likely to contribute to *increases* in migration toward coastal areas in Bangladesh by the end of the century. In that setting, coastal populations’ ability to move is constrained by access to cash and the relative affordability of livelihood alternatives to migration^28^.

Physical infrastructure can also influence the impacts of natural hazards on human mobility, particularly for isolated communities on island states. While populations of small-island states can seek shelter in other areas of the island, movement to other regions of an island can be constrained by limited road coverage. In addition, many small island states have limited, time-constrained, and expensive off-island transportation options. As island road networks are often dictated by topography and commonly positioned close to flatter areas along the coastline or other water bodies, evacuations are more likely to be hampered by inundations, landslides, and other disruptions to infrastructure that affect road access^29,30^. Even in the ‘large-island’ case of the Japanese Tohoku earthquake, tsunami, and nuclear meltdown at Fukushima, the evacuation was made difficult or impossible at scale in the short term due to damage to coastal roads and infrastructure^31^.

### Political, legal, and bureaucratic drivers

**Fig. 1:**
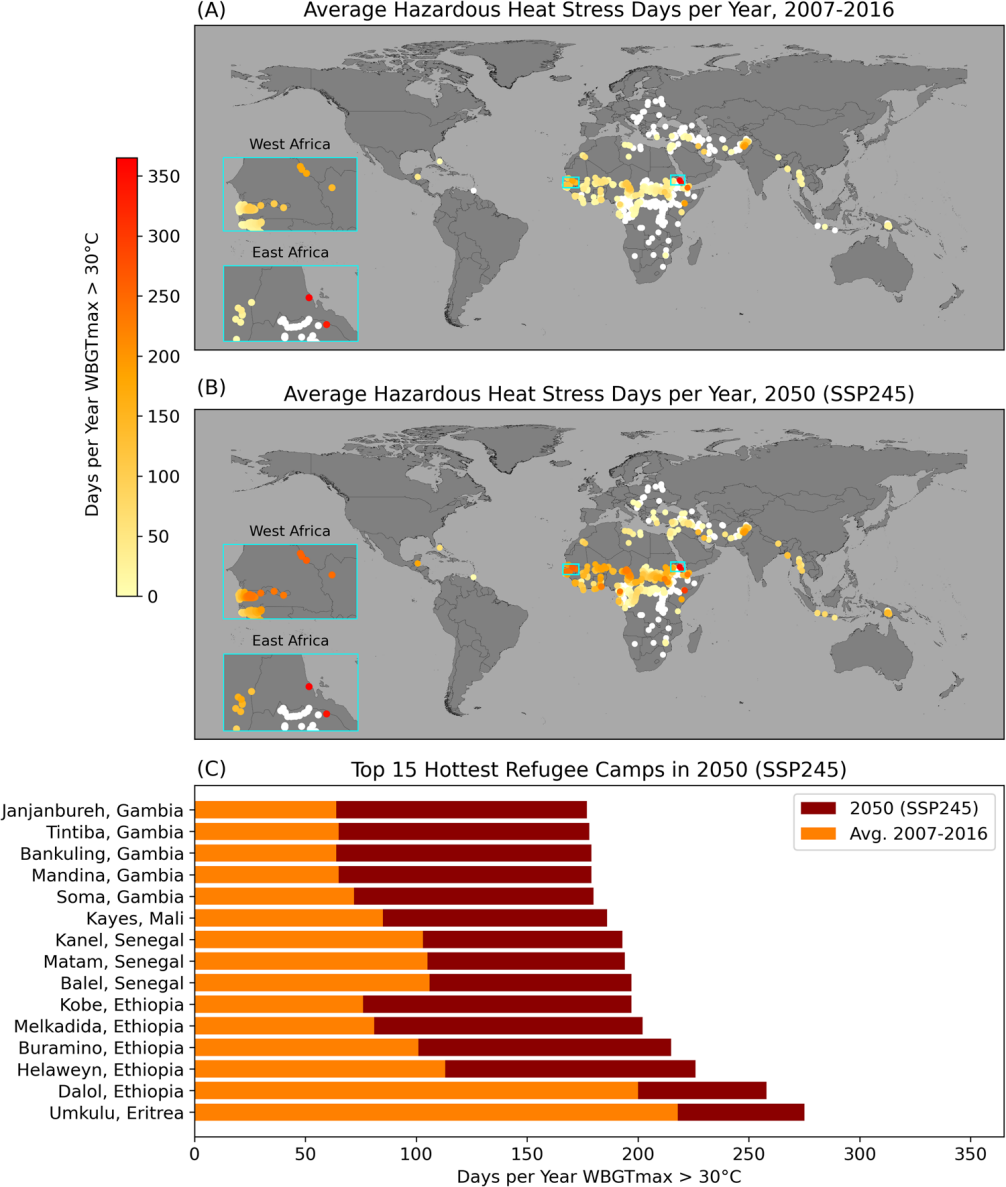
Heat-impacted refugee camps. Location of refugee camps where (**A**) the average number of days per year shaded daily maximum wet bulb globe temperature (WBGT_max_) exceeded 30 °C for 2007–2016. **B** is the average number of days WBGT_max_ exceeded 30 °C for 2050 following SSP245 (middle of the road) radiative forcing climate projection. **A**, **B** White indicates zero days per year WBGT_max_ >30 °C. **C** lists the top 15 hottest refugee camps in 2050 under SSP245, with the count days in 2050 vs. the 2007–2016 baseline. The cyan boxes in (**A**) and (**B**) are the approximate locations of the camps identified in (**C**). WBGT_max_ >30 °C is the ISO standard^80^ for risk to occupational heat stress for acclimated people under moderate metabolic rates (65–130 Wm^−2^). For context, a monthly average WBGT of 30 °C has been associated with increased mortality rates among vulnerable populations^43^. The UNHCR People of Concern and the CHC-CMIP6 datasets are published under a Creative Commons Attribution 4.0 International Public License; see https://creativecommons.org/licenses/by/4.0/.

International borders, as well as restrictions on internal mobility, can impose long-term immobility. On the international front, in the face of increasing natural hazards and risks from SLC with additional warming, populations living on island states lack legal mechanisms enabling them to leave and thus find themselves unable to move abroad^30^. Similarly, refugees living in host countries with restrictive asylum policies may be physically confined to a camp or specific areas of a country with minimal or no ability to migrate post-hazard or when confronting slow-onset climate impacts (Fig. 1). These policy restrictions on mobility compound the extreme poverty and resource deprivation faced by many refugees and the dwindling availability of humanitarian aid, the delivery of which is itself subject to political will, reprioritization, and climate-related disruptions.

A striking example of political and legal barriers to migration within a country is the case of the Rohingya refugee population in Bangladesh, forced to flee Myanmar after a years-long campaign of ethnic cleansing. This case, like many others, presents substantial heterogeneity in migration aspirations and capabilities across groups impacted by climate change^32^. In addition, Bangladesh has periodically resorted to forced relocation to keep refugees in specific areas of the country, with differentiated risks and impacts of natural hazards^33^. Most refugees in the country reside in Cox’s Bazar District, where refugee settlements are among the most densely populated areas in the world^34^. Many camps face high levels of landslide exposure, while others face a high risk of coastal and inland flooding^35,36^. Climate change impacts could further diminish migration opportunities while making the consequences of immobility increasingly catastrophic.

Refugees are not the only group facing barriers to migration due to entry restrictions. Many migrants find themselves stuck in transit countries and forced to survive on marginal lands. Evidence from North Africa and Mexico indicates that stranded migrants are particularly vulnerable to flooding^37–39^. Because of language and cultural barriers, and limited access to humanitarian assistance, these populations face exceptionally high exposure to natural hazards. By way of example, the COVID-19 pandemic substantially worsened the situation for migrants traveling through Mexico, with many left stranded after the enactment of US Title 42, which prevented their entry to the United States ostensibly in view of limiting the spread of the disease. Confined to Mexico, these migrants faced significant natural hazards there, including exposure to floods in the Matamoros camp for asylum-seekers along the US-Mexico borders in the summer 2020^40–42^.

As an illustration of climate change impacts, nearly all current refugee camps will face an unprecedented rise in hazardous heat stress (Fig. 1). By 2050, the hottest fifteen camps may experience nearly 200 or more days of hazardous heat stress per year under middle-of-the-road climate projections (SSP245). Should these locations continue to house refugees with little investment in adaptations like air conditioning, such heat stress is likely to drastically harm health and well-being among refugees, with recent evidence suggesting a precipitous rise in mortality^43^. Heat stress is just one of multiple natural hazards projected to worsen for involuntary immobile groups worldwide^44^.

### Social and cultural drivers

Access to social services, networks, attachment to place, and cultural symbolism also drive involuntary immobility. Indigenous communities and residents in informal or deprived areas of cities face social and cultural barriers and are systematically predisposed to a lack of services and financial opportunities^45^. These dynamics exist both in high-income and lower- and middle-income communities and countries ^46^.

For instance, Pacific Island states face displacement situations that break community connections, including losses and disconnection from culture, language, and religious ties. In addition to bureaucratic barriers, the ability to migrate can be limited by attachment to their lands^47,48^. In Aotearoa (New Zealand), indigenous Māori communities face potential relocation of coastal *marae*, and communal meeting grounds of cultural and religious significance due to recurrent flooding, soil erosion, and SLC. Yet, the consequences of potentially losing the links to “ancestral lands” for the maintenance of Māori communities, combined with the lack of available land and land ownership restrictions, have contributed to hurdles in relocating *marae* to safer places^49^.

Unhoused populations (UPs) face exposure to natural hazards without sufficiently reliable options to avoid risk. While some UPs, such as those living in shelters, may be able to move about a defined area, the extent to which they can find adequate refuge from threats remains limited, especially when the distribution and area of risk are changing^50^ In New York City, UPs face diverse social barriers that limit their ability to find shelter when facing floods, storms, and heat waves. For example, heat-related mortality among UPs is projected to double by 2050, while a paucity of data exists for similar analyses for other natural hazards^51^. As a consequence, UPs are more likely to suffer from high temperature and air pollution, relative to populations having access to housing^51^. While there may be warning systems for poor air quality and extreme temperature, UPs are often unable to relocate to safer locations, underpinning the need for tailored policy responses^52^.

## Principles for research and policy responses

A more nuanced representation of involuntary immobility leads to a more appropriate governance discourse in Latin America and the Pacific Islands^53^. However, gaps remain in the development of both international and local policies and services tailored specifically for immobile populations in the context of climate change. While acknowledging that policies in response to involuntary immobility in the context of climate change will have to be grounded locally and co-developed with affected populations, we can nevertheless draw some guiding principles. From general to specific research and policy responses, these include the following.

### Incorporate aspirations and capabilities in climate and disaster-risk policy

For policies and services intending to address climate-related risks, the outputs will be incomplete at best if the needs, aspirations, and capabilities of populations facing immobility are not central. People’s aspirations will need to be taken into account to understand whether immobility is involuntary or voluntary. If people want to leave but cannot, policies should aim at reducing drivers of immobility, i.e., barriers to migration. Suppose populations hope to stay where they live now. In that case, the policy approaches should focus on enabling people to sustain their livelihoods in situ in a safe and human rights-conforming manner.

The development of policy responses to involuntary immobility in the context of climate change requires conducting a systematic review of affected communities and their ability to mitigate the impact of natural hazards and climate change, to adapt in situ, or to relocate, with specific attention to those communities that may be hard to reach or otherwise marginalized^54^. Losses and damages from climate change can make it difficult to re-establish life and community, particularly without community support. Increasingly, the importance and benefit of integrating people’s mobility aspirations and choice to stay is acknowledged^55,56^, which could inform more appropriate policy responses^57^.

Legal drivers of involuntary immobility that prevent specific populations from moving within countries, among them refugees, should be abolished to mitigate the impact of climate change on these vulnerable populations. The same applies to small-island states, whose populations have few realistic prospects to stay over the long term in the face of SLC. While we recognize the challenges inherent in easing barriers to international travel, at minimum, policies should be implemented in a way that prevents migrants from being left stranded in transit or in conditions that adversely affect their safety^53^.

### Include drivers of involuntary immobility in data collection processes

The lack of recognition of involuntary immobility within the international institutions that set the policy agendas for climate migration and displacement has repercussions for data on involuntary immobility^3,58–60^. While explicitly including immobile populations within pre-disaster, early warning, and anticipatory action remains challenging due to issues in observing immobility before natural hazards materialize, the lack of data on locations and aspirations of populations itself continues to be a major constraint in policy and risk management^61^.

In assessing and planning data collection on immobility, local and national authorities, international organizations, and NGOs should evaluate the type of natural hazards they are confronting and, on this basis, consider how these hazards might affect mobility patterns. This type of assessment must be conducted for both rapid and slow-onset hazards, and for compound, cascading, and complex ones. For instance, when examining sudden-onset hazards, immobile populations are likely to comprise populations that do not have access to necessary transport to evacuate, for example, UPs or poor households, or are prevented from doing so, for example, incarcerated people. When confronting slow-onset natural hazards, immobile populations may include those households that lack the resources to cover migration costs to find alternate livelihoods, including the potential costs of learning a new trade in the case of rural-urban migration. Knowledge of local stakeholders and experts is key in identifying these groups of populations at risk of immobility.

Once the type of vulnerable populations has been identified, a mapping exercise should be conducted to identify the size and location of these populations for a given natural hazard. Such tools could include assessments based on feedback from local communities and experts, in conjunction with available demographic data, for example, from censuses, or the use of surveys to investigate the aspiration of respondents to move and their capacity to do so^53^. This latter tool is likely vital for slow-onset natural hazards, which may entail long-distance migration and for which at-risk populations are potentially larger and more complicated to identify. We emphasize our first point that whenever possible, care should be taken to incorporate people’s aspirations to move, particularly when working with marginalized populations such as Indigenous groups, and note that even when co-produced, biases will likely be present that may influence the information shared on preferences and aspirations. As an example from the Rohingya-encamped population context, preferences to anticipate and react in the face of climate risk in ways that demanded a significant deviation from daily activities differed significantly across sub-camps. Perceived risk and prioritized actions were influenced by a variety of factors, including elders and religious leaders. However, the individual risk perception of climate shocks can still play an essential role in selecting and timing an action^34,62^.

### Co-production and data on subgroups of involuntary immobile populations

The types of populations at risk of immobility vary substantially across contexts and would benefit from data-related co-production tailored to the population and drivers of involuntary immobility in the context of climate change. The lack of detailed population data on marginalized populations should not limit governments and institutions from supporting and striving for policy and disaster planning that includes immobile populations. Moreover, the availability and accessibility of appropriate and sufficient data require an integration of science into policy translation processes to avoid the risk of prioritizing some populations over others^63,64^.

Certain immobile groups, such as inmates or UPs, are likely missed by census data used in vulnerability analyses^65,66^. In addition, disagreement across some population datasets could create differences in policy implementation, including but not limited to prioritizing resources for climate change adaptation and activation of early warning systems^67^. Ethical considerations need to involve an assessment of data quality to ensure that the most vulnerable people are not left out of policy responses and that action prioritizes those most in need. Furthermore, high-resolution data are needed to meet community needs, justice, and equity to be met^68,69^. Investing in data collection processes that are co-produced with people facing an immobility risk together with decision-makers is a key step in outlining and addressing these data challenges. Then, the integration, translation, and decision-making steps need to reflect both the data from the co-production process as well as other lessons learned from this process^70^.

### Establish a global involuntary immobility support mechanism

Finally, we stress the need for international climate and disaster-risk policies to reduce the risk of involuntary immobility by improving mobility in some cases and by improving in situ conditions in others. We advocate the creation of an international mechanism that would support policy initiatives and develop guiding principles for the data collection on involuntary immobility for low- or middle-income countries that may lack the resources to appropriately address these situations^53^. In doing so, international institutions relevant to climate migration and displacement would obtain an explicit mandate to consider involuntary immobility, unlocking additional climate action. While the shape of such a mechanism or body could take different forms, it could take a similar shape to the UN Office of the Special Advisor on Solutions to Internal Displacement.

Immobile populations are at risk of suffering severe impacts resulting from natural hazards and compound, cascading, or complex disasters^10,20,71,72^. Even under optimistic climate projections, climate change impacts will continue to harm involuntarily immobile populations if substantial policy shifts and targeted investment in adaptation are not enacted.

Centering involuntary immobility in climate and disaster-risk policy and research enables a more nuanced understanding of how climate change disproportionately affects vulnerable populations. This understanding is essential for recognizing the drivers and reducing associated risks leading to involuntary immobility in the first place^20^. It further illuminates the gaps in current climate adaptation strategies, highlighting the need for appropriately designed policies that address these unique challenges. We aimed to bridge these gaps, advocating for a collaborative approach to climate action that prioritizes the protection and empowerment of all individuals through our focus on involuntary immobility. By doing so, we seek to align our involuntary immobility research with worldwide efforts to address climate and environmental-related risks, underscoring the imperative for scientifically and ethically sound policies.

## Methods

### World cafe

We used a World Cafe approach as a participatory action research technique to discuss research and policy implications of involuntary immobility in the context of climate change. The World Cafe method has been applied to leverage knowledge on complex topics and is a well-vetted technique from the field of applied decision science^73,74^. The World Cafe event was held in June 2023 with 53 participants from policy and research during the 2023 Managed Retreat Conference at Columbia University. This session aimed to highlight immobility in the context of environmental, political, and social drivers, and to uncover underlying themes of involuntary immobility in the context of climate change. The World Cafe consisted of three phases: (1) discussion sessions, (2) a group deliberation, and (3) the development of a synthesis manuscript co-authored by selected participants.

#### Discussion sessions

The first phase of the World Cafe consisted of three small group discussion rounds on the themes of (i) drivers and impacts, (ii) challenges and obstacles, and (iii) policy implications and guardrails. Each table consisted of 8–9 participants and one facilitator serving a dual role: guiding the discussion and taking notes. Three graduate students assisted in taking notes. After each round, participants were encouraged to change tables.

#### Group deliberation

The second phase of the World Cafe was a group deliberation. The deliberation combined a participatory summary of the World Cafe session through facilitators and a discussion around unique learnings (e.g., *What surprised you most about today’s discussions?*) and points not raised during the World Cafe (e.g., *What was missing from the discussions today?*).

#### Synthesis manuscript

Nine participants expressed interest in developing a synthesis manuscript with the five World Cafe session leads (L.T., A.K., F.C., C.H., C.T.) and subsequently came to form the authoring group.

On the basis of notes of the World Cafe session and the group deliberation, anonymized transcripts were prepared with points raised and then summarized. The summarized transcripts from the World Cafe rounds were analyzed by L.T., A.K., and F.C. through qualitative content analysis with support from C.H. and C.T. Everyone in the author group contributed to the writing of this paper.

### Refugee-heat stress observations and projections

Refugee camp locational data are based on publicly available “People of Concern” data from UNHCR^75^ accessed on 21 February 2024. We filtered the UNHCR dataset to 1344 refugee population locations across spontaneous locations (931), planned settlements (384), unplanned settlements (5), and dispersed locations (24). We exclude refugee population locations in Lebanon, which are recorded differently than other countries, and exclude locations of asylum-seeking populations, internally displaced persons, returnees, stateless populations, and those classified as unknown.

We use the Climate Hazards Center Coupled Model Intercomparison Project Phase 6 climate projection dataset (CHC-CMIP6) to estimate historical (2007–2016) and future (2050) average annual heat stress at the location of all refugee camps worldwide. Available at 5 km globally, CHC-CMIP6 is among the highest-resolution climate projections specifically designed to estimate future heat stress in data-sparse refugee-hosting regions like the African Sahel^76^.

A highly accurate 5-km blended satellite and station-derived daily maximum air temperature record—CHIRTS-daily^77^—and down-scaled relative humidity estimates from ERA5 climate reanalysis form the foundation of the CHC-CMIP6 daily shaded daily maximum wet bulb globe temperature (WBGT_max_) observational record. Next, coarse-grained (100–250 km) CMIP6 ensembles for 245 and SSP 585 scenarios are used to develop high-resolution (0.05°) 2030 and 2050 “delta” fields, which is the difference between CMIP6 multi-model ensemble-based changes between 1983–2016 and 2025–2035 and 2045–2055. The delta fields are then used to perturb the WBGT_max_ observational record to derive the CHC-CMIP6 SSP245 and SSP 585 2030 and 2050 projections. We use the SSP245 projections for 2050, derived from the CHC-CMIP6 dataset, because its high spatial resolution better captures local climate heterogeneity than the coarse-grained CMIP6 projections, for a full description of CHC-CMIP6^76^.

To measure heat stress at refugee camp locations, we first estimate the average annual number of days per year WBGT_max_ exceeded 30 °C from 2007 to 2016 using the CHC-CMIP6 observational record. We then estimate future heat stress as the average annual number of days per year WBGT_max_ exceeded 30 °C from 2007–2016 under a 2050 climate following SSP245. In other words, we use the CHC-CMIP6 data to adjust the daily observed weather from 2007–2016 based on the projected radiative forcing for SSP245 in 2050. While we fully recognize that a plethora of heat stress metrics exist^78^, we estimate heat stress as the number of days where WBGT_max_ exceeded 30 °C because it is the International Organization for Standardization (ISO) criteria for occupational risk to heat stress for acclimated people under moderate metabolic rates (65-130 Wm^-2^). These ISO criteria are well-established and commonly used in the heat health literature^43,65,79^. Monthly average WBGT of 30 °C has been associated with increased mortality rates among vulnerable populations^43^.

## Data Availability

Refugee camp locations are available in the “UNHCR People of Concern” dataset https://data.unhcr.org/en/geoservices/. The CHC-CMIP6 is available 10.21424/R47H0M. Code to reproduce Fig. 1 is publicly available on Github: https://github.com/cascadet/refugee-heat.
